# Gastric tuberculosis mimicking submucosal tumor: a case series

**DOI:** 10.1186/s12876-020-1175-x

**Published:** 2020-01-30

**Authors:** Rong Zhu, Yuankun Zhou, Haibo Wang, Lianjun Di, Kui Zhao, Biguang Tuo, Huichao Wu

**Affiliations:** Department of Gastroenterology, Affiliated Hospital, Zunyi Medical College, Zunyi, 563003 People’s Republic of China

**Keywords:** Gastric tuberculosis, Submucosal tumor, Diagnosis, Endoscopic ultrasonography

## Abstract

**Background:**

Gastric tuberculosis mimicking submucosal tumors is extremely rare and often misdiagnosed.

**Case presentation:**

Three cases of gastric tuberculosis mimicking submucosal tumors were identified among patients admitted to local county hospitals because of upper abdominal discomfort and pain, with gastroscopy showing gastric submucosal tumors. The patients were admitted to our hospital for endoscopic ultrasonography (EUS). As EUS revealed evidence of tuberculosis for the first two patients, we first considered gastric tuberculosis. However, because of atypical characteristics of the miniature ultrasonic probe and EUS, the lesion in the third patient was misdiagnosed as a stromal tumor, and the patient underwent endoscopic submucosal dissection (ESD), and intraoperative characteristics and postoperative pathology confirmed the lesion to be tuberculosis.

**Conclusions:**

EUS is of great value in the diagnosis of gastric tuberculosis, especially gastric tuberculosis mimicking submucosal tumors. Here, we summarize some significant characteristics of gastric tuberculosis by EUS, which will be of substantial value to clinical work.

## Background

Gastric tuberculosis is quite rare, and its clinical manifestations are often not specific. Therefore, the diagnosis of gastric tuberculosis is very difficult, and it is often misdiagnosed. In particular, gastric tuberculosis mimicking submucosal tumors is more likely to be misdiagnosed than other types of gastric tuberculosis [1]. Endoscopic ultrasonography (EUS) is an important diagnostic tool for submucosal tumors. Here, we report 3 interesting cases of gastric tuberculosis mimicking submucosal tumors and summarize some common characteristics of gastric tuberculosis based on EUS; we believe that these features will be very important for the diagnosis of gastric tuberculosis.

## Case presentation

The 3 patients involved were born and raised in rural areas of Guizhou Province, China, and had never left the province. They were admitted to local county hospitals because of upper abdominal discomfort and pain; gastroscopy showed gastric submucosal tumors in each. The patients were then admitted to our hospital for endoscopic ultrasonography (EUS). A miniature ultrasonic probe (UM-2R 12 MHz; Olympus, Tokyo, Japan) with a circular scanning ultrasound endoscope (UE260-AL5, Olympus, Tokyo, Japan) was used to visualize both the gastric wall and adjacent structures, such as the para-gastric or abdominal lymph nodes.

### Case 1

**Fig. 1:**
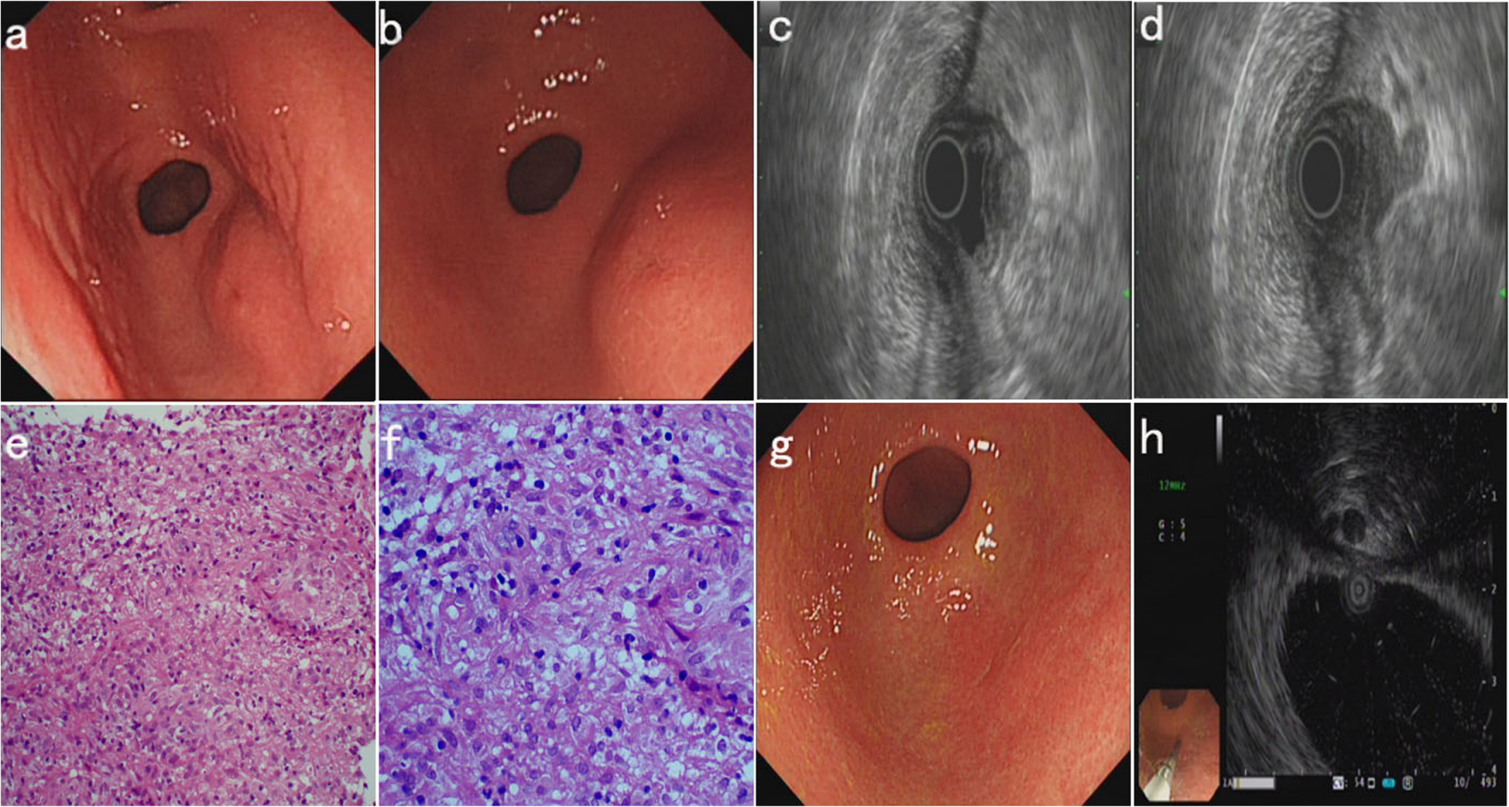
Endoscopic and pathological findings of case 1. **a** (far view) and **b** (near view), Gastroscopy: a mucosal protrusion with a slightly depressed central area of the gastric antrum. **c** and **d**, EUS: hypoechoic lesions of the gastric antrum (**c**), associated with enlarged lymph nodes outside the gastric wall (**d**). **e** (200X) and **f** (400X), Histopathological examination (HE staining): chronic granulomatous inflammation. **g**, Gastroscopy after treatment: a white mucosal depression similar to an ulcer scar. **h**, EUS after treatment: the hypoechoic lesions and enlarged lymph nodes had disappeared

The patient is a 25-year-old woman of Han ethnicity who had upper abdominal discomfort and distension. The patient had no other symptoms and no previous history of other diseases. Gastroscopy revealed a mucosal protrusion of the posterior wall of the gastric antrum, with a slightly depressed central area (Fig. 1a, far view and Fig. 1b, near view). EUS revealed hypoechoic lesions (14.8 mm X 20.8 mm), with irregular boundaries and nonhomogeneous inner echo; the gastric wall was thickened and deformable, and the five-layer structure had disappeared. Enlarged lymph nodes were observed outside the gastric wall and hypoechoic lesions were associated with the lymph nodes (Fig. 1c and d). Repeated deep excavation biopsy along the central depression and histopathological examination (HE staining) revealed chronic granulomatous inflammation (Fig. 1e 200X and Fig. 1f 400X). Therefore, we considered the lesion to be tuberculosis, and we performed erythrocyte sedimentation rate (ESR) (45 mm/h) and tuberculin purified protein derivative (PPD) (+) analyses, chest computed tomography (CT) (no obvious abnormalities), abdominal ultrasonography (ascites and some enlarged lymph nodes), an ascites test (exudate, ADA: 45 U/L), and colonoscopy (no tuberculosis or other lesions). The patient was diagnosed with gastric tuberculosis and tuberculous peritonitis and administered regular anti-tuberculosis drug treatment. One year later, the patient’s gastric tuberculosis and ascites had completely subsided. Based on gastroscopic examination, the gastric mucosal protrusion of the posterior wall of the gastric antrum had disappeared, and a white mucosal depression that resembled an ulcer scar had formed (Fig. 1g). EUS showed that the hypoechoic lesions in the posterior wall of the gastric antrum had disappeared, the wall of the stomach was thinner, and the enlarged lymph nodes outside the gastric wall had also disappeared (Fig. 1h).

### Case 2

**Fig. 2:**
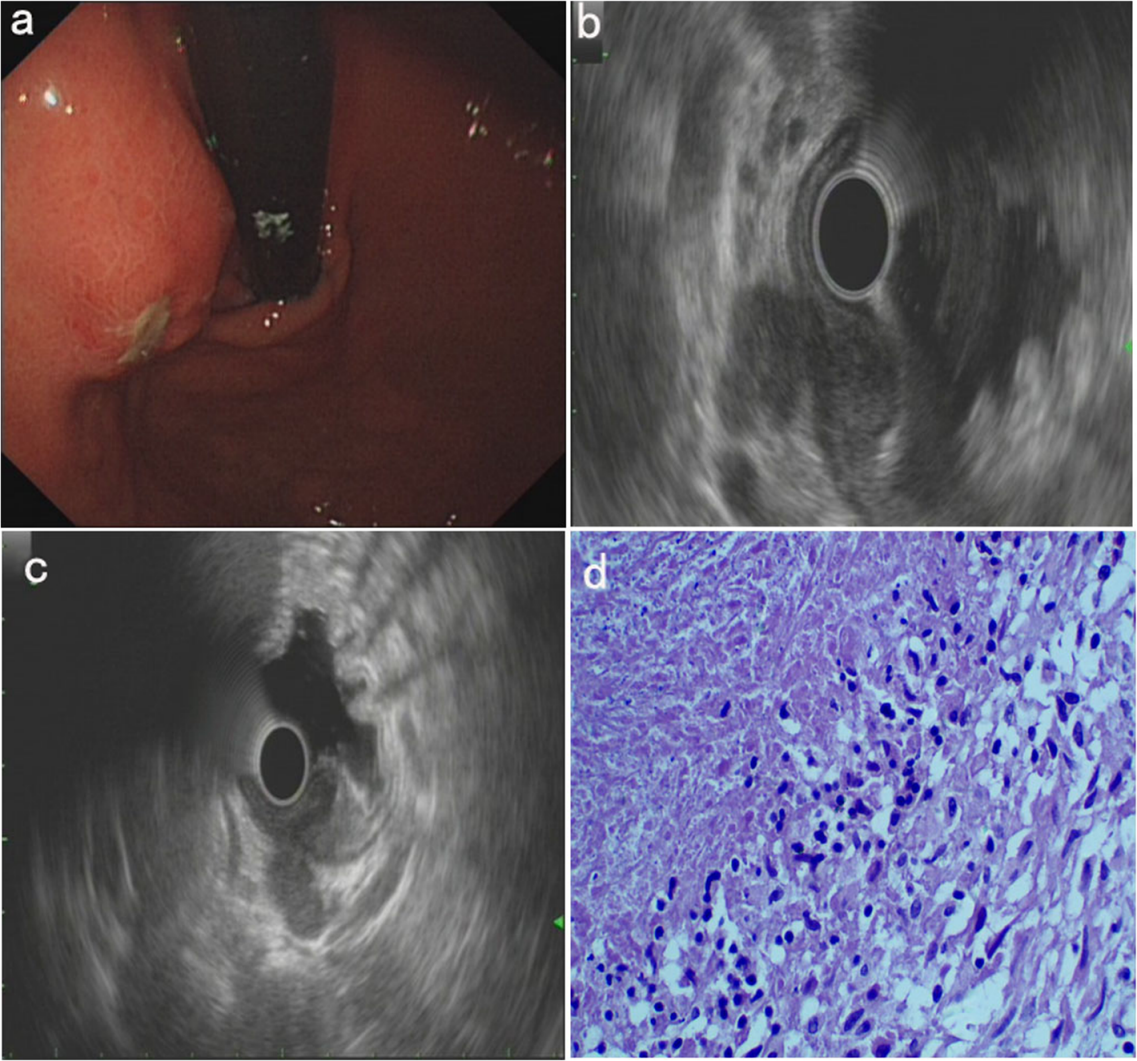
Endoscopic and pathological findings of case 2. **a**, Gastroscopy: a mucosal protrusion with central erosion of the fundus ventriculi. **b** and **c**, EUS: hypoechoic lesions of the fundus ventriculi (**b**), associated with enlarged lymph nodes outside the gastric wall (**c**). **d**, Histopathological examination (HE staining, 400X): chronic granulomatous inflammation with caseous necrosis

The patient is a 52-year-old woman of Miao ethnicity, and she had experienced upper abdominal swelling and pain for 6 months. She had lost 3 kg of weight over the previous 2 months. In the previous week, she had a low fever (axillary temperature: 37.5–38.0 degrees centigrade) and night sweats. Ten years prior, she had pulmonary tuberculosis and was cured. There was no previous history of other diseases. Gastroscopy revealed a mucosal protrusion with central erosion of the fundus ventriculi (Fig. 2a). EUS showed hypoechoic lesions (20.2 mm X 25.0 mm), with irregular boundaries and nonhomogeneous inner echo, a poorly structured and thickened gastric wall, and enlarged lymph nodes outside the gastric wall (Fig. 2b). Moreover, the hypoechoic lesions were associated with lymph nodes (Fig. 2c). Repeated deep excavation biopsy along the central erosion and histopathological examination (HE staining) demonstrated chronic granulomatous inflammation with caseous necrosis, which is consistent with tuberculosis (Fig. 2d). By combining her history, EUS and pathological findings, the patient was diagnosed with gastric tuberculosis. We also conducted ESR analysis (50 mm/h), PPD (+), chest CT (some calcified spots), and abdominal ultrasonography (some enlarged lymph nodes in the upper abdomen). The patient was given standard anti-tuberculosis treatment for 13 months and had a good response.

### Case 3

**Fig. 3:**
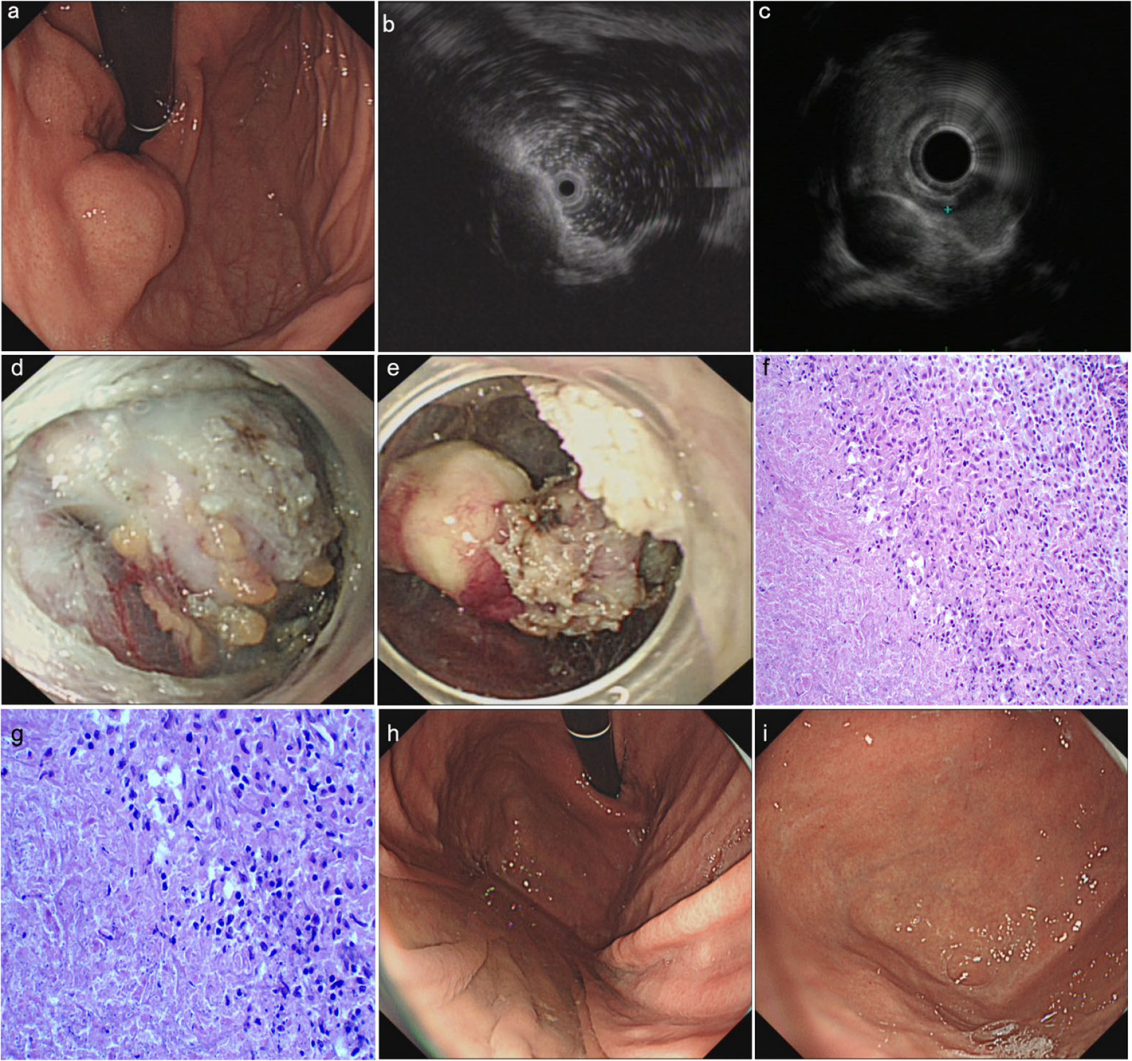
Endoscopic and pathological findings of case 3. **a**, Gastroscopy: a mucosal protrusion with a smooth surface in the fundus of the stomach. **b** and **c**, Miniature ultrasonic probe and EUS: a hypoechoic lesion, suspected of originating from the muscularis propria of the gastric wall. **d** and **e**, ESD: the lesion with complete packaging and multiple nodules on the surface, continuous with a lump similar to the enlarged lymph node outside the gastric wall. **f** (200X) and **g** (400X), Histopathological examination (HE staining): chronic granulomatous inflammation with caseous necrosis. **h** (far view) and **i** (near view), Gastroscopy after treatment: a slight white mucosal depression with scar formation

The patient was a 16-year-old boy of Han ethnicity who was a local high school student; he had experienced upper abdominal pain and fatigue for 3 months. At the time of admission to our hospital, he had lost 4 kg in weight. He had no other symptoms and no previous history of other diseases. Gastroscopy revealed a mucosal protrusion with a smooth surface in the fundus of the stomach (Fig. 3a). A miniature ultrasonic probe and EUS revealed a hypoechoic lesion (10.8 mm X 22.4 mm) that was suspected of having originated from the muscularis propria of the gastric wall; the internal echo was uniform, and the boundary was clear (Fig. 3b and c). Based on our experience and these findings, we considered the lesion to be an interstitial tumor. After communicating with the patient and his family, they decided to choose minimally invasive surgery, namely, endoscopic submucosal dissection (ESD), under general anaesthesia. After completing the preoperative examination (chest CT: obsolete pulmonary tuberculosis and abdominal ultrasonography: no obvious abnormalities), we performed ESD on the patient. During the operation, we did not find any lesions originating from the muscularis propria. However, after opening the muscularis propria, we were surprised to find a lesion with a relatively complete package and multiple nodules on the surface. This lesion severely compressed the muscularis propria and pushing it into the gastric lumen, and it was continuous with a lump similar to the enlarged lymph node outside the gastric wall (Fig. 3d and e). After the lesion was successfully removed, a histopathological examination (HE staining) was routinely performed. Surprisingly, the pathological results revealed chronic granulomatous inflammation with caseous necrosis, which is consistent with tuberculosis (Fig. 3f 200X and Fig. 3g 400X). We next performed ESR (35 mm/h) and PPD (+) analyses. The patient was eventually diagnosed with gastric tuberculosis and given anti-tuberculosis treatment for half a year, at which point the lesion in the fundus of the stomach had disappeared, leaving a slight white mucosal depression with scar formation (Fig. 3h and i).

## Discussion and conclusions

Tuberculosis is a worldwide problem that seriously threatens human health, including in China. Tuberculosis is mostly found in economically underdeveloped areas with poor living conditions, and Guizhou Province is a high-incidence area of tuberculosis in China [2]. Here, we report some rare cases of gastric tuberculosis.

Gastric tuberculosis is quite rare and often secondary, and its mechanism of infection is still not fully understood. The possible modes of infection leading to gastric tuberculosis [3] are as follows: (1) *Mycobacterium tuberculosis* is swallowed and directly invades the gastric mucosa. (2) *M. tuberculosis* invades the gastric wall through the blood or lymphatic system. (3) Tuberculosis in the lymph nodes or peritoneum around the stomach spreads directly to the stomach. Based on EUS, the first two patients we report herein had enlarged lymph nodes outside the gastric wall, and the lymph nodes were continuous with gastric tuberculosis. In the third patient, although we did not find lymph nodes associated with the lesion on EUS and misdiagnosed it as an interstitial tumor, intraoperative examinations and postoperative pathology confirmed that the lesion was tuberculosis; the lesion was also continuous with the enlarged tubercular lymph nodes outside the gastric wall. Therefore, tuberculosis in the lymph nodes around the stomach that spreads directly to the gastric wall is a very important means by which gastric tuberculosis can develop.

The clinical manifestations of gastric tuberculosis are often not specific [4]. Upper abdominal discomfort or pain is the most common symptom; some patients have tuberculous blood symptoms, and a few patients may have upper gastrointestinal bleeding [5] or pyloric obstruction [6, 7]. All 3 patients we report herein were admitted for upper abdominal discomfort or pain.

The diagnosis of gastric tuberculosis is very difficult because the clinical manifestations, upper gastrointestinal contrast and gastroscopy findings are not specific. Indeed, it is often misdiagnosed as chronic ulcers [8], gastric cancer [9–11], or other conditions [12]. A definitive diagnosis is based on a positive biopsy or bacteriological examination. However, because the lesions in gastric tuberculosis are often located below the mucosa, the biopsy positivity is very low, especially for gastric tuberculosis mimicking submucosal tumors. Nonetheless, EUS may be helpful for diagnostic purposes. Interestingly, for the above-mentioned 3 patients with gastric tuberculosis mimicking submucosal tumors, we identified some common, characteristic EUS manifestations, providing very important guidance for the diagnosis of gastric tuberculosis. These signs are as follows: (1) a thickened, deformable, poorly defined gastric wall structure; (2) hypoechoic lesions on the stomach wall, with irregular boundaries and nonhomogeneous inner echo; (3) enlarged para-gastric or abdominal lymph nodes; and (4) connection between the stomach wall lesion and the enlarged para- gastric or abdominal lymph nodes. Of these four characteristic manifestations, the fourth is especially important because it suggests that tuberculosis in the para-gastric or abdominal lymph nodes around the stomach has spread directly to the stomach. Because the first two patients exhibited all four characteristics, we initially diagnosed them as having gastric tuberculosis. However, for the third patient, because of the relatively complete package and severely compressing muscularis propria that was protruding into the gastric lumen, the miniature ultrasonic probe and EUS showed that the lesion was likely to have originated from the muscularis propria. Thus, we initially misjudged it to be an interstitial tumor. When we performed ESD, we found that the lesion was continuous with a lump similar to the enlarged lymph node outside the gastric wall, and postoperative pathology ultimately confirmed tuberculosis.

For suspected gastric tuberculosis, multiple deep biopsies, repeated biopsies, EUS-guided fine-needle aspiration or EUS-guided fine-needle biopsy (EUS-FNA or EUS-FNB) [13] should be performed. The first two patients underwent repeated deep excavation biopsy along the central erosion of the lesions and received a final diagnosis. For the third patient, we should have observed the lesion completely at all levels and investigated its surrounding conditions more carefully, especially by examining the posterior field of the lesion by EUS. In addition, further abdominal CT scans may be helpful to reveal the relationship between the lesion and the surrounding structures, such as the para-gastric or abdominal lymph nodes, and to exclude the diagnosis of some tumors. When considering suspected tuberculosis, we FNA or FNB should be performed to obtain tissue for pathological diagnosis.

Overall, this article emphasizes that EUS is of great value for diagnosing gastric tuberculosis, especially gastric tuberculosis mimicking submucosal tumors. When considering tuberculosis, it is also very important for medical staff to take effective measures to protect themselves.

## Data Availability

All data analysed during this study are included in this manuscript.

## Abbreviations

ADA: Adenosine deaminase
CT: Computerized tomography
ESD: Endoscopic submucosal dissection
ESR: Erythrocyte sedimentation rate
EUS: Endoscopic ultrasonography
EUS-FNA: Endoscopic ultrasonography-guided fine-needle aspiration
EUS-FNB: Endoscopic ultrasonography-guided fine-needle biopsy
PPD: Purified protein derivative

## References

[1] Kim SH, Park JH, Kang KH (2005). Gastric tuberculosis presenting as a submucosal tumor. Gastrointest Endosc.

[2] Chen W, Yang L, Yang H (2016). Is tuberculosis health education reaching the public in China? A cross-sectional survey in Guizhou Province. BMJ Open.

[3] Amarapurkar DN, Patel ND, Amarapurkar AD (2003). Primary gastric tuberculosis – a report of 5 cases. BMC Gastroenterol.

[4] Udgirkar S, Surude R, Zanwar V, Chandnani S, Contractor Q, Rathi P (2018). Gastroduodenal Tuberculosis: A Case Series and Review of Literature. Clin Med Insights Gastroenterol.

[5] Nasa M, Kumar A, Phadke A, Sawant P (2016). Hematemesis: unusual presentation of isolated gastric tuberculosis. Indian J Tuberc.

[6] Arabi NA, Musaad AM, Ahmed EE, Ibnouf MMAM, Abdelaziz MSE (2015). Primary gastric tuberculosis presenting as gastric outlet obstruction: a case report and review of the literature. J Med Case Rep.

[7] Padmanabhan H, Rothnie A, Singh P (2013). An unusual case of gastric outlet obstruction caused by tuberculosis: challenges in diagnosis and treatment. BMJ Case Rep.

[8] Chetri K, Prasad KK, Jain M, Choudhuri G (2000). Gastric tuberculosis presenting as non-healing ulcer: case report. Trop Gastroenterol.

[9] Eray İC, Rencüzoğulları A, Yalav O (2015). Primary gastric tuberculosis mimicking gastric cancer. Ulus Cerrahi Derg.

[10] Yaita H, Nakamura S, Kurahara K (2014). Gastric tuberculosis resembling depressed type early gastric cancer. Endoscopy.

[11] Kim S-E, Shim K-N, Yoon SJ (2006). A case of gastric tuberculosis mimicking advanced gastric cancer. Korean J Intern Med.

[12] Liu PF, Chang CS, Wang J, Wu CC, Yeh HZ (2009). Primary gastric tuberculosis. Endoscopy.

[13] Sharma V, Rana SS, Gunjan D, Chhabra P, Sharma R, Bhasin DK (2015). Primary gastric tuberculosis mimicking a submucosal tumor. J Dig Endosc.

